# Surgical Techniques for Abdominoperineal Resection for Rectal Cancer: One Size Does Not Fit All

**DOI:** 10.3389/fsurg.2022.818097

**Published:** 2022-02-24

**Authors:** Simon Wilkins, Raymond Yap, Shehara Mendis, Peter Carne, Paul J. McMurrick

**Affiliations:** ^1^Cabrini Monash University Department of Surgery, Cabrini Hospital, Malvern, VIC, Australia; ^2^Department of Epidemiology and Preventive Medicine, School of Public Health and Preventive Medicine, Monash University, Melbourne, VIC, Australia; ^3^Department of Oncology Research, Cabrini Hospital, Malvern, VIC, Australia; ^4^Colorectal Unit, Department of Surgery, Alfred Hospital, Melbourne, VIC, Australia

**Keywords:** Colorectal Cancer, surgical technique, surgical outcome, extra-levator abdominoperineal excision (ELAPE), abdominoperineal resection

## Abstract

Abdominoperineal resection (APR) of rectal cancer is associated with poorer oncological outcomes than anterior resection. This may be due to higher rates of intra-operative perforation (IOP) and circumferential resection margin (CRM) involvement causing higher recurrence rates and surgical complications. To address these concerns, several centers advocated a change in technique from a standard APR to a more radical extra-levator abdominoperineal excision (ELAPE). Initial reports showed that ELAPE reduced IOP rates and CRM involvement but increased wound complications and longer surgical duration. However, many of these studies had unacceptable rates of IOP and CRM before retraining in ELAPE. This may indicate that it was a sub-optimal surgical technique, which improved upon training, that had influenced the high CRM and IOP rates rather than the technique itself. Subsequent studies demonstrated that the CRM involvement rate for ELAPE was not always lower than for standard APR and, in some cases, significantly higher. The morbidity of ELAPE can be high, with studies reporting higher adverse events than APR, especially in terms of wound complications from the larger perineal incision required in ELAPE. Whether ELAPE improves short- or long-term oncological outcomes for patients has not been clearly demonstrated. The authors propose that all centers performing rectal cancer surgery audit surgical outcomes of patients undergoing APR or ELAPE and examine CRM involvement, IOP rates, and local recurrence rates, preferably through a national body. If rates of adverse technical or oncological outcomes exceed acceptable levels, then retraining in the appropriate surgical techniques may be indicated.

## Introduction

The use of abdominoperineal resection (APR) as a surgical treatment for rectal cancer has declined over the last three decades as treatment paradigms have evolved (1, 2). As a result of technological advances in stapling techniques and adjuvant therapy, sphincter preservation by anterior resection and anastomosis rather than APR has become increasing performed for even the most distal rectal tumors (3, 4). However, APR remains a necessary surgical technique in specific settings where sphincter preservation is impossible due to oncological or functional reasons. Due to the requirement of a permanent stoma and the resultant perineal wound, APR is a procedure with significant morbidity. In addition, patients undergoing APR are associated with worse oncological outcomes and higher recurrence rates than low anterior resection despite appropriate adjuvant therapy (1, 5, 6).

To achieve total mesorectal excision through an APR procedure is technically demanding. The technical difficulty in achieving clear circumferential resection margins (CRM) and avoiding intra-operative perforation (IOP) in the lower rectum is one of the main reasons cited for poorer oncological outcomes due to higher rates of local recurrence compared with patients undergoing low anterior resection (7, 8). A reduction in the mesorectal tissue at the pelvic floor with a tendency toward “waisting” of the mesorectum at the level of the puborectalis muscle increases the difficulty in obtaining a clear CRM and avoiding IOP (9–11). CRM involvement and IOP are implicated in increased local recurrence rates and worse oncological outcomes for patients (5, 7, 12). Therefore, much work has been done to improve and audit the outcomes from these surgical techniques to avoid these technical pitfalls, and this is explored below.

## A Change in Technique?

In order to minimize CRM involvement and rates of IOP, several centers in Europe have advocated for a change in technique from a standard APR to the more radical extra-levator abdominoperineal excision (ELAPE) (5, 9, 10, 13, 14). The differences between APR and ELAPE begin with the abdominal component of the surgery, where dissection of the mesorectum from the pelvic floor is restricted. With the ELAPE technique, the perineal approach is performed with the patient in the prone jack-knife position, and the levator ani muscles are divided under direct vision as far laterally as possible, close to obturator internus, as opposed to standard APR, where it is usually done in the lithotomy position, and the levator ani resected close to the rectum. The ELAPE approach results in a wide excision with a “cylindrical” excision of anorectum and mesorectum (5, 9). Using this technique, authors have reported a reduction in CRM involvement and IOP compared with standard APR (9, 13, 14). A systematic review by Stelzner et al. (14) demonstrated a significant reduction in rates of intra-operative perforation (4.1 vs. 10.4%) and CRM involvement (9.6 vs. 15.4%) for ELAPE vs. standard APR. In addition, they reported lower local recurrence rates in the ELAPE group (6.6 vs. 11.9%).

## Drawbacks of the New Technique

Had there been no pitfalls to this more radical surgical approach, the argument for change would be easily won. However, there are many potential adverse outcomes with ELAPE, which were apparent from the outset. Some studies have described poor outcomes with standard APR with CRM involvement rates up to 49.6%, IOP rates of 28.2%, and a 17.9% local recurrence rate (9). If these published figures for standard APR were reflected in high-volume centers worldwide, then a rapid change in technique to ELAPE would be warranted everywhere. The evidence that ELAPE reduces CRM involvement is mixed. CRM rates were higher in ELAPE than APR (15.9 vs. 7%), and in fact, the risk of CRM was significantly higher among ELAPE patients in a Danish study (15). By comparison, a similar, multi-centre Spanish study demonstrated near-identical positive CRM rates in APR and ELAPE procedures (13.1 vs. 13.6%) (16). A systematic review of seven studies totalling 2,672 patients concluded that the current evidence did not indicate ELAPE was statistically superior over standard APR in terms of positive CRM and IOP (17). The Swedish Colorectal Cancer Registry found ELAPE had a higher positive CRM rate than APR (10 vs. 6%), whereas IOP rates were higher in APR than ELAPE (11 vs. 8%) (18). A further study in Sweden showed positive CRM rates lower in ELAPE than APR (17 vs. 20%) but higher IOP rates in ELAPE than APR (13 vs. 10%) (19). The results from these Swedish studies may be a reflection of selection bias, with ELAPE cases being larger, more difficult tumors.

It was not unexpected that ELAPE patients have wound complications, with a larger defect requiring more complex closure. The original paper advocating ELAPE described the perineal phase with a gluteus maximus flap and that the perineal phase be assisted by one or two plastic surgeons to reconstruct what was described as the “gaping musculocutaneous defect” in Holm et al. (20) and Kennelly et al. (21). Although this has not been investigated in relation to ELAPE specifically, prone positioning (part of the ELAPE technique) carries its own set of risks described in other surgical procedures. These include airway complications (endotracheal tube displacement), oropharyngeal swelling, acute glaucoma, optical compartment syndrome, cardiovascular compromise and neurological injuries (22). These problems with ELAPE were acknowledged and a subsequent modification of the ELAPE technique, termed laparoscopic transabdominal ELAPE, removed the need for position change to a prone position and so reduced patient morbidity and operating times (23).

If ELAPE did not represent higher morbidity compared to APR, then a change in technique, even if it did not produce clearly better oncological outcomes, could be considered appropriate. However, previous studies using the ELAPE approach have demonstrated significant disadvantages, including a longer operating time (an increase of 2 h over standard APR), related to the extra time required for a change in patient position and the need to create a flap repair to reconstruct the larger defect from the more extensive resection (9), as well as increased wound complications associated with an extra-levator approach (9). Some ELAPE studies have reported wound complications rates of up to 46.6% (24). Comparison of APR and ELAPE at a Swedish hospital found higher adverse results for ELAPE compared to APR in terms of perineal wound infection (46 vs. 28%) and perineal wound revision (22 vs. 8%) (19).

## Achieving the Best Oncological Outcome

Whether ELAPE is a superior oncological technique over standard APR has also been much debated. A Danish nationwide database study concluded that resection of rectal cancers by ELAPE did not improve short-term oncological results when compared to standard APR (15). Nonetheless, these results would suggest that the choice of technique is not a primary factor in influencing CRM or IOP rates, nor in any subsequent oncological outcomes. More critical may be the need for adequate training and sufficient surgeon volume, which the focus on ELAPE may be producing, and the real underlying cause behind the perceived improvement in oncologic outcomes (11). A study of 163 patients at a single specialist center undergoing standard APR demonstrated a CRM involvement of 3.7 % (6 cases) and an intra-operative perforation rate of 1.2% (2 cases) (25). These rates are lower than many previous published series of patients using either standard APR or ELAPE and compare favorably to multi-centre and single-site studies (25).

Thus, there remains significant conjecture as to the superiority of ELAPE over standard APR in the routine treatment of low rectal cancer where sphincter preservation is not possible or practical. Accordingly, there have been suggestions toward a more selective approach and a word of caution on the widespread use of ELAPE. The Danish study quoted above suggested that specific patients should be “selected” for ELAPE as it was a technique that did not seem suitable for routine use (15). This was echoed by work from Gina Brown's group in the UK, which suggested that ELAPE be reserved for selected cases only depending on tumor height and location, such as advanced lateral tumors (and therefore advanced stage) close to or at the level of the pelvic floor. It was also concluded that it would be wrong to oversimplify ELAPE as an oncologically superior operation but rather one that should be considered in a tailored approach toward surgery (26). A study from MD Anderson Cancer Center supports this notion where they used a tailored approach to either APR or ELAPE dependent on patient and tumor factors (advanced pathological stage, stage-appropriate chemoradiotherapy) and demonstrated an involved CRM rate of only 1.6% in a study of 128 patients (27).

## Discussion

In summary, while ELAPE was reported to have resulted in a reduction in the rates of adverse surgical outcome after APR in many studies (involved CRM, IOP and local recurrence rate), in many of these series, the baseline levels of these adverse outcomes were unacceptably high, indicating deficiencies in surgical technique. Under these circumstances, the change to ELAPE may well reflect a re-training process that has resulted in improvements in surgical outcome that is independent of the original technique. If acceptable rates of these parameters are being carefully audited and achieved, then a change in technique or retraining in the relevant surgical approach may not be necessary.

We propose that all centers undertaking abdominoperineal resection of rectal cancer should participate in a prospective audit of surgical outcomes, including the three parameters of involved CRM, IOP, and local recurrence rate, supported by a quality improvement program. The assessment of clinically relevant quality measures can be enhanced with national bodies, such as National Accreditation Program for Rectal Cancer (NAPRC) in the USA (28). Indeed, if individual centers are achieving rates of involved CRM, intraoperative perforation, and local recurrence rates at acceptably low levels, then it is likely that their technique is already sound. If rates of adverse outcomes exceed acceptable levels in a persistent fashion, then a discussion regarding retraining or a change in technique is indicated. This may involve retraining in standard APR and/or, for certain selected cases, ELAPE. The focus should not always be on a particular surgical technique but producing the best oncological and quality of outcomes for rectal cancer patients. Anatomical factors in relation to the position of the tumor could influence as to whether ELAPE or APE may be more appropriate. Tumors wholly within the anal canal or early stage tumors where the excision margins are not threatened may lend themselves toward an APE approach. ELAPE may be more technically feasible where the access to the low pelvis is anticipated to be poor i.e., the obese male with a narrow pelvis. One size does not fit all, and the surgical technique employed should be tailored for each patient and each tumor.

## Conclusions

We have discussed the advantages and disadvantages of ELAPE and standard APR and examinded whether one approach is superior to the other in terms of oncological outcomes. We conclude that the case for the superiority of ELAPE has not been demonstrated, and improvements seen with ELAPE may be due to the re-training process rather than a superior technique. We propose that all centers using ELAPE and standard APR should audit surgical outcomes of these procedures. If rates of adverse outcomes exceed acceptable levels, then retraining in the appropriate surgical technique may be indicated. A tailored surgical approach should be taken for each patient.

## Data Availability Statement

The original contributions presented in the study are included in the article/supplementary material, further inquiries can be directed to the corresponding author.

## Author Contributions

SW, RY, SM, PC, and PM were involved in the initial planning of the article, the drafting of the manuscript, and approval of the final version to be submitted.

## Conflict of Interest

The authors declare that the research was conducted in the absence of any commercial or financial relationships that could be construed as a potential conflict of interest.

## Publisher's Note

All claims expressed in this article are solely those of the authors and do not necessarily represent those of their affiliated organizations, or those of the publisher, the editors and the reviewers. Any product that may be evaluated in this article, or claim that may be made by its manufacturer, is not guaranteed or endorsed by the publisher.
